# Olive Fungal Epiphytic Communities Are Affected by Their Maturation Stage

**DOI:** 10.3390/microorganisms10020376

**Published:** 2022-02-05

**Authors:** Joana Castro, Daniela Costa, Rui M. Tavares, Paula Baptista, Teresa Lino-Neto

**Affiliations:** 1 Centre of Molecular and Environmental Biology (CBMA), Department of Biology, University of Minho, Campus of Gualtar, 4710-057 Braga, Portugal; jdsecastro@gmail.com (J.C.); danielacosta@bio.uminho.pt (D.C.); tavares@bio.uminho.pt (R.M.T.); 2 Centro de Investigação de Montanha (CIMO), Instituto Politécnico de Bragança, Campus de Santa Apolónia, 5300-253 Bragança, Portugal; pbaptista@ipb.pt

**Keywords:** *Olea europaea* L., anthracnose, fruit ripening, olive cultivar, organic production, integrated production

## Abstract

The phyllosphere comprises the aerial parts of plants and is colonized by a great diversity of microorganisms, either growing inside (as endophytes) or on the surface (as epiphytes) of plant tissues. The factors that structure the diversity of epiphytes and the importance of these microorganisms for host plant protection have been less studied when compared to the case of endophytes. In this work, the epiphytic fungal communities from fruits of the olive tree (olives) in different maturation stages (green and semi-ripened), obtained from different olive orchard managements (integrated and organic production) and from distinct cultivars displaying different susceptibilities to olive anthracnose (*Cobrançosa* and *Madural*), are compared by using a metabarcoding approach. We discuss whether such differences in host resistance against anthracnose depend on both the fungal taxa or fungal community composition. A total of 1565 amplicon sequence variants (ASVs) were obtained, mainly belonging to the Ascomycota phylum and Saccharomycetes class. Although significant differences on epiphytic fungal richness were observed among olives obtained in different production systems and maturation stages, these factors in addition to host cultivar did not influence the composition of the epiphytes. Despite these results, a co-inertia analysis showed that *Aureobasidium* spp. and Sporocadaceae spp. were positively associated with the green olives of the cv. *Madural* produced under integrated production, while Saccharomycetales spp. (*Kluyveromyces*, *Candida*, *Kazachstania* and *Saccharomyces*) were positively associated with the semi-ripened olives of the cv. *Cobrançosa* obtained from organic production. The discriminant power of these fungi, some of them recognized as biocontrol agents, suggest that they might be important in conferring differences on host plant susceptibility to anthracnose.

## 1. Introduction

The olive tree (*Olea europaea* L.) is cultivated in distinct world geographical regions for the production of olives for table consumption and oil production. In the Mediterranean region, where 95% of world olive groves are located, this ancient culture has a great economic, social and ecological importance [1]. During the last decades, the traditional olive production has been replaced by intensive and super-intensive cultivation systems [2], due to the increasing international demand for olive oil and table olives [1]. However, these production systems are not compatible with sustainable agriculture (organic and integrated productions), which are the pillars of the European Model for Agriculture, according to the Directive 2009/128/EC. Both organic and integrated productions system are, in general, associated with the presence of high biodiversity, and thus provide several important ecosystem services when compared to the intensive and super-intensive olive groves [3]. For instance, there is increasing evidence showing that plants with greater diversity of microorganisms are more protected from diseases due to, for example, a higher occurrence of microbial antagonists [4,5,6]. Indeed, a diverse microbial community has been recognized to inhabit the aerial parts of plants (phyllosphere), being able to grow both epiphytically (on the surface of plant tissues) or endophytically (within the tissues) [7]. These microorganisms can reduce the pathogenic infection of plant tissues, either directly through antibiosis or resource competition, or indirectly by the induction of plant resistance responses [8]. More recently, the overall microbial community that is naturally associated with plants has been recognized as affecting the infection ability of pathogens and even disturbing disease progression [9]. More important than the one-pathogen–one-disease concept, the scientific community is becoming aware of the importance of all pathobiome, in which the combined effect of microbial communities, host and environment plays an important role in host responses and/or conditions [10]. Therefore, olive-phyllosphere-associated microorganisms have been explored to search for biocontrol agents and to design new strategies for the control of olive diseases (e.g., [11,12,13]). Among diseases, olive anthracnose, caused by *Colletotrichum* spp., has been considered as one of the most destructive olive diseases worldwide, being responsible for olive production losses of 80–100% [14]. Although some olive cultivars are more prone to infection than others, anthracnose-resistant olive tree genotypes have not yet been described [14]. For example, the cv. *Cobrançosa* is less susceptible to anthracnose than the cv. *Madural* [8]. This disease is known to affect the aerial parts of olive trees, causing damage mostly to the fruits [13].

Taking into account the importance of microbial communities in affecting olive diseases, a metabarcoding approach is used in this work to evaluate the effect of olive maturation (green and semi-ripened), agricultural management (organic and integrated productions), as well as olive genotype (cvs. *Cobrançosa* and *Madural*), in olive epiphytic fungal microbial diversity. Aspects related to potential implication of fungal composition on host plant susceptibility to anthracnose are discussed in the context of developing sustainable control strategies against this disease.

## 2. Materials and Methods

### 2.1. Olive Fruit Sampling

Sampling was performed in two olive orchards located in Mirandela (northeast of Portugal; 41°36′28.6″ N 7°13′26.7″ W and 41°29′31.1″ N 7°15′28.2″ W). Each orchard comprised olive trees from two distinct olive cultivars (*Cobrançosa* and *Madural*). Although exhibiting identical edapho-climatic conditions, both orchards were under different management practices (organic or integrated production) for at least 10 years. The organic orchard is not irrigated, ploughed only to a shallow depth (10 cm) two times per year, and only copper-based products were applied once per year. The second orchard has been managed under integrated pest management guidelines [15]. In this orchard, one treatment with spinosad against olive fruit fly and three treatments with copper-based products were performed during the study year. Tillage was performed three times per year, and no irrigation was performed. From each orchard, five olive trees of each cultivar were randomly selected and were used for sampling apparently healthy olive fruits. Olives were sampled at two different maturation stages, in September 2019 (green: fruit epidermis yellowish green) and October 2019 (semi-ripened: epidermis shows red spots in less than half of the fruit), whose determination was based on Beltrán et al. (2008) [16]. The collected samples were individually placed into sterile bags, kept at 4 °C, and processed within two days. All olives were observed with a stereo microscope (Leica S9 D, Leica Microsystems, Wetzlar, Germany) to ensure that fruits did not have any injury.

### 2.2. Preparation of DNA from Fungal Epiphytes

A total of 5 replicates from each condition (olive maturation stage, management practice, and cultivar) were prepared, resulting in a total of 40 samples (5 replicates × 2 maturation stages × 2 production systems × 2 olive cultivars). To each replicate (25 g of olives), 50 μL of a peptone water solution (10 g/L peptone, 5 g/L sodium chloride) with 0.1% (*v*/*v*) Tween 20 were added and olives were shaken at 200 rpm for 2 h. The supernatant was centrifuged at 4500× *g* and the obtained pellet was kept at −80 °C, until DNA extraction. All steps for extracting DNA from olive epiphytes were performed at room temperature, except when clearly stated otherwise. The epiphytic genomic DNA was extracted through an adaptation of the Ahmadi et al. protocol [17]. The pellet was suspended in 1 mL of phosphate buffer solution (1×, pH 6.7) and was continuously mixed for 3 min at 25 °C, at 160 rpm. Then, the suspensions were centrifuged at 400× *g* for 5 min. The supernatant was collected and centrifuged at 8000× *g* for 15 min. After centrifugation, the pellet was resuspended in 500 μL of suspension buffer (10 mM Tris–HCl pH 8.0; 1 mM EDTA; 20 mg/mL lysozyme; 30 μL proteinase K (20 mg/mL)), transferred to a new tube and incubated at 37 °C for 30 min. The mixture was treated with 500 μL of lysis buffer (100 mM Tris–HCl pH 8.0; 50 mM EDTA; 0.5 M NaCl; 4% SDS; 2% PVPP) and kept at 60 °C, for 30 min, with intermittent mixing at every 5 min. To each tube, 250 μL potassium acetate (5 M, pH 5.5) and 250 μL phenol/chloroform (25:24) was added and mixed by inversion. The collection of the aqueous phase was performed after centrifugation at 3000× *g* for 8 min. The extracted DNA was precipitated by adding 60 μL of sodium acetate (3 M, pH 5.2) and two volumes of absolute ethanol, followed by incubation for 3 min. The final DNA precipitate was pelleted at 14,000× *g*, for 10 min, and eluted in 40 μL of ultra-pure water.

### 2.3. Fungal ITS2 Amplification and Sequencing

To determine DNA integrity and suitability for sequencing, DNA obtained from the collected samples was amplified, using the universal fungal primers *ITS3*-*ITS4* for the *ITS2* region [18]. PCR reactions were conducted in a total volume of 10 µL, containing 0.2 U/μL *NZYTaq II 2x Green Master Mix* (NZYtech, Lisboa, Portugal), 10 mM of each primer, 3 μL of ultra-pure water and 1 μL of extracted DNA. Negative controls comprised reactions with water replacing template DNA. PCR reactions were performed in a *MJ Mini* (BioRad, Hercules, CA, USA) thermocycler, using the following protocol: initial denaturation at 94 °C for 5 min, followed by 35 cycles of 94 °C for 50 s, 45 °C for 30 s, 72 °C for 90 s, with a final extension at 72 °C for 5 min. PCR products were run on a 1% (*w*/*v*) agarose gel. As three DNA samples (1-PM1, 1-PM3 and 1-AM2) were not amplifiable by PCR, they were not sequenced. All the others resulted in a fragment of the expected size (340–415 bp).

The quantity of purified DNA was determined using a fluorescent DNA quantification assay with *dsDNA BR (Broad Range) Assay Kit* (ThermoFisher Scientific, Waltham, MA, USA). DNA-specific fluorescence was then detected with a *Qubit 3.0 Fluorometer* (ThermoFisher Scientific, Waltham, MA, USA). Fungal epiphytic communities were assessed by metabarcoding (*Illumina MiSeq* system, Illumina, San Diego, CA, USA), through paired-end sequencing (2 × 250 bp) for the *ITS2* region, using a sequencing service provider (Genoinseq, Coimbra, Portugal). For sequencing purposes, a pool of forward primers *ITS3NGS1_F* (5′-CATCGATGAAGAACGCAG-3′), *ITS3NGS2_F* (5′-CAACGATGAAGAACGCAG-3′), *ITS3NGS3_F* (5′-CACCGATGAAGAACGCAG-3′), *ITS3NGS4_F* (5′-CATCGATGAAGAACGTAG-3′), *ITS3NGS5_F* (5′-CATCGATGAAGAACGTGG-3′), and *ITS3NGS10_F* (5′-CATCGATGAAGAACGCTG-3′) and the reverse primer *ITS4NGS001_R* (5′-TCCTSCGCTTATTGATATGC-3′) [19] were used.

### 2.4. Processing of Sequencing Data

Raw reads were extracted from *Illumina MiSeq* system (Illumina) in fastq format and the quality report of each library sequencing was viewed in *FastQC* version 0.11.8 [20]. The sequences trimming was performed in *Sickle* [21], using the default parameters. For correcting errors in reads before merging, the Bayeshammer module from *SPAdes* package [22] was used. The *Usearch* version 8.0.1623 [23] was used for merging the overlapped paired-end reads and for further quality filtering. The sequence size filtering was then performed by *fastq-mcf* from the *ea-utils* package [24]. The *micca* version 1.7.0 software pipeline [25] was used (i) for loading the datasets into a single FASTQ file, (ii) for discarding sequences displaying an expected error rate greater than 1%, (iii) for assigning similar sequences to Amplicon Sequence Variants (ASVs)—UNOISE3 protocol—and removing chimeric sequences, and (iv) for assigning taxonomy to each sequence with a reference database for fungi (UNITE database version 8.0) [26]. Sequences of unclassified ASVs were further searched using NCBI-BLAST [23] for understanding their taxonomic classification. ASVs that were not assigned to any taxonomic level (unclassified reads) were not included in further analysis. Due to the reduced number of classified fungal sequences (699) found in 2-PC3 sample, this replicate was excluded from further analyses to avoid the underestimation of community structure and diversity. Since differences in sampling depth were detected and for mitigating biases, all datasets were subsampled using *QIIME* 1.9.0 [27] for an even number of sequences (3526 reads, found in 2-PM2 sample).

### 2.5. Diversity of Fungal Epiphytes

The diversity of fungal epiphytes was determined in olive fruit samples at different maturation stages, production systems and olive cultivars. The diversity was assessed by evaluating the richness (number of ASVs) and by computing Simpson’s (1-*D*) and Shannon–Wiener (*H*’) diversity indices [28,29] with *PAST3* [30]. *PAST3* was also used for computing rarefaction curves with individual-based rarefaction. All diversity indices and estimators are presented as the mean of replicates (3–5), displaying respective SE values. Data were evaluated by analysis of variance (ANOVA) and means were compared using Tukey’s post hoc test (*p* ≤ 0.05), performed using *GraphPad Prism 7.00* (GraphPad Software, La Jolla, CA, USA). Non-metric multidimensional scaling (NMDS) was carried out to explore the similarity of fungal communities between maturation stages (green vs. semi-ripened), production systems (organic vs. integrated) and cultivars (*Cobrançosa* vs. *Madural*). NMDS was carried out with Bray–Curtis dissimilarity coefficients, using square root transformation of data. This coefficient was used to quantify the compositional dissimilarity between samples, taking into consideration the presence/absence and abundance of fungal taxa. The model’s goodness of fit was measured by 2D stress level, where a good representation is considered when 2D < 0.2. Analysis of similarity (ANOSIM) was used to test significant differences between fungal communities. The *R* value generated by ANOSIM gives the degree of discrimination between groups and ranges from -1 (similar communities) to 1 (completely dissimilar) [18]. NMDS and ANOSIM were performed using *Community Analysis Package v. 4.0* [31]. For co-inertia analysis (CIA), only the epiphytic fungal ASVs with the greatest power to separate semi-ripened from green, organic from integrated production and cv. *Cobrançosa* from cv. *Madural* olives were used. To select these fungal ASVs, a random forest analysis was computed with the *RandomForest* package [32] from *RStudio*. The importance of fungal ASVs to distinguish communities was measured according to their Gini coefficient value (the higher the value, the greater its importance) [33]. To find a co-structure between the three sets of variables that are linked by the same individuals [34], a co-inertia analysis (CIA) was performed using the *co-inertia()* function of *ade4* package [35] in *RStudio*. The *table.value()* function to visualize the results.

## 3. Results and Discussion

A set of 1,935,982 raw reads was generated from 37 olive fruits samples, ranging from 2449 to 116,690 raw reads per sample (in 2-PC3 and 2-AM3 samples, respectively; Table S1). After quality evaluations, a total of 1,170,598 high-quality sequences (processed reads, Table S1) were recovered. Almost 43% of total processed reads were classified as fungal taxa (1565 ASVs), the remaining being identified as plant sequences (1033 ASVs) or unclassified taxa (458 ASVs). To diminish the potential bias introduced by different sequencing depths from distinct samples, the dataset was subsampled to the least number of fungal sequences found in a single sample (3526 sequences; 2-PM2 sample). After subsampling, fungal classified sequences were identified up to genus level in most of the cases (62%; Table S1). Only few fungal ASVs (16%) were identified to species level, which could be related to the limited (or even inexistent) genetic variation within *ITS* regions among closely related species [36]. This is particularly true for Ascomycota phylum members [36], which is the most representative phylum in this study. The high number of unclassified ASVs (458) and unidentified families (26% of all assigned ASVs) suggests the existence of several rare or still unknown olive-tree-associated fungal taxa, as previously suggested by Fernández-González [37] and reported by us [38].

### 3.1. Epiphytic Fungal Community Diversity in Olives from Different Conditions

**Figure 1:**
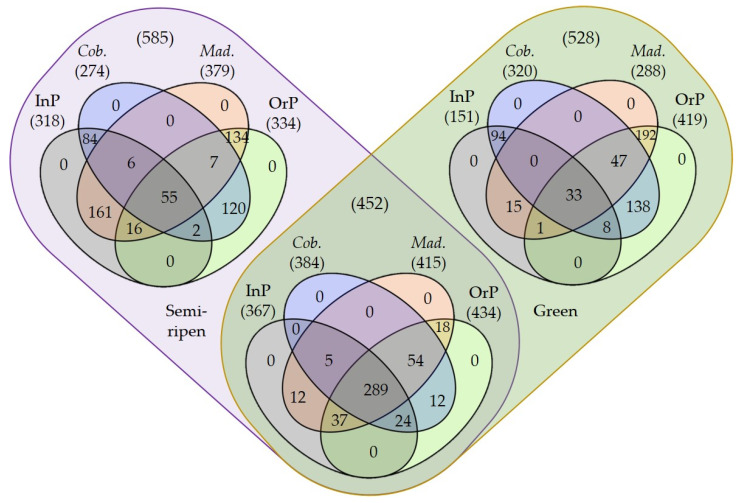
Venn diagrams of shared and exclusive epiphyte fungal ASVs present in green olives and/or semi-ripened olives, regarding the production system (integrated production (InP) and/or organic production (OrP)) and olive cultivar (*Cobrançosa* (*Cob.*) and/or *Madural* (*Mad.*)). Numbers in brackets refer to the number of shared/exclusive ASVs.

The fungal community diversity among olive maturation stages, production systems and cultivars was compared by computing the corresponding rarefaction curves and determining the richness (*S*) and diversity indices (Simpson’s (1-*D*) and Shannon’s (*H*’)). The rarefaction curves suggested that all fungal communities were well represented and the sampling effort was enough to disclose the fungal structure of olives obtained in the studied conditions (Figure S1). In any case, microbial communities from olives obtained through integrated production management were better represented, as more samples achieved an almost saturation plateau than those produced by organic management (Figure S1A,D). Accordingly, this later management system led to a higher number of identified ASVs (1187) than integrated production management (836), in particular in green olives that displayed 853 vs. 518 ASVs, respectively (contrasting with 768 vs. 685 ASVs in semi-ripened olives, respectively; Figure 1, Table S1). A higher epiphytic richness in the organic production of olives, when compared to integrated production (Table 1), suggests that conditions in organic farming are more favorable for developing a richer fungal epiphyte community. These results have also been reported for the epiphytic fungal communities (determined using culture-dependent methods) of apple fruits produced by organic and integrated production systems [39]. This may be due to the great rate of application of copper-based products in integrated production, which limits fungal development and richness, unlike what happens in organic production. Accordingly, the negative impact of copper-based fungicide application on epiphytic yeasts and yeast-like communities of grape berries has been previously demonstrated [40]. Significant richness (*S*) differences were also detected among the microbial communities from olives at different maturation stages, although exhibiting contrasting trends according to the olive production system used (Table 1, Figure 1). Green olives exhibited a higher microbial richness under organic production (853) than semi-ripened olives (768), but these later presented a higher richness following an integrated production (685) than green olives (518). Interestingly, these findings were more evident for cv. *Madural* olives (671 OrP-Green, 610 OrP-Semi-ripened, 392 InP-Green, 581 InP-Semi-ripened; Figure 1), as cv. *Cobrançosa* olives exhibited more similarly enriched communities (605 OrP-Green, 563 OrP-Semi-ripened, 453 InP-Green, 465 InP-Semi-ripened; Figure 1). Despite this, there was no statistical significance in richness regarding olive cultivar within the same production system, as both cultivars are under the same environmental conditions and due to the already reported environmental effects in the structure of epiphyte communities [41]. This result is concordant with previous reports of epiphytic fungal communities using culture-dependent methods in olives [42] and in mango fruits from different cultivars [43]. Differences detected in richness parameter (*S*) were not directly translated into significant differences in other diversity parameters, such as Simpson’s (1-*D*) and Shannon’s (*H*’) indexes (Table 1), suggesting a relative evenness of microbial community abundances. Dissimilar values of *H’* (although not significantly different) suggest a differential contribution of dominant species in detected diversity of distinct samples.

Many biotic and abiotic factors are currently described to shape the phyllosphere microbiota, including environmental variables, season, soil management systems, host genotype (cultivar) and geographical location [41,44]. To evaluate the influence of abiotic factors in olive fungal communities, a cluster analyses (two-dimensional NMDS), as well as ANOSIM and PERMANOVA were performed. NMDS based on Bray–Curtis dissimilarity indexes (that considers the presence/absence and abundance of species) resulted in a good representation (2D stress < 0.2) of olive epiphyte fungal communities (Figure S2). However, when using Jaccard’s coefficient (based only on the presence/absence of species), a good representation of communities was lacking (2D stress was higher than 0.2), revealing the importance of ASVs abundance in the discrimination of these fungal communities. In any case, NMDS ordinations did not differentiate fungal communities from distinct maturation stages, production systems or cultivars (Figure S2). These results were corroborated by ANOSIM that did not reveal relevant differences among the overall olive fungal communities from distinct production systems (*R* = 0.002, *p* > 0.05) or cultivars (*R* = −0.001, *p* > 0.05), although a statistically significance was found for olive maturation stages (*R* = 0.06, *p* ≤ 0.05) (Table S2). The same results were obtained using PERMANOVA analyses that revealed that 5% of the variation of fungal epiphyte communities present in olives were explained by olive maturation (PERMANOVA; *F*_1,35_ = 1.800, *R*^2^= 0.050, *p* < 0.05). Production system and cultivar factors were not relevant to explain epiphytic fungal structure (PERMANOVA; *F*_1,35_ = 0.831, *R*^2^= 0.024, *p* > 0.05 (for production system); *F*_1,35_ = 1.1654, *R*^2^= 0.033, *p* > 0.05 (for cultivar)). The importance of maturation stage to structure endophytic fungal communities in olives has been reported by using culture-dependent methods [45], but there have been no reports about the importance of maturation stage in epiphytic community structure. Despite that, the influence of maturation stage on epiphytic fungal communities has been demonstrated in grape berries [40,46] and mango fruit [47]. Additionally, our results are in line with a previous suggestion that host genotype is not relevant in shaping epiphytic fungal communities [42]. Accordingly, in a more extensive study (using 290 isolated fungal operational taxonomic units), the major drivers shaping the epiphytic composition were found to be the season, wind speed and temperature [41].

Taken together, although significant differences on microbial richness were observed among microbial communities from olives obtained in different production systems and maturation stages, there was not evident dissimilar fungal communities among olives obtained in such conditions. The existence of dominant ASVs and the presence of multiple rare ASVs could have reduced the detected variation in community structure between samples. Indeed, despite the high number of identified ASVs (1565), few accounted for the majority of reads (23% of ASVs were represented by only five or less reads).

### 3.2. Epiphytic Fungal Community Structure in Olives from Different Conditions

**Figure 2:**
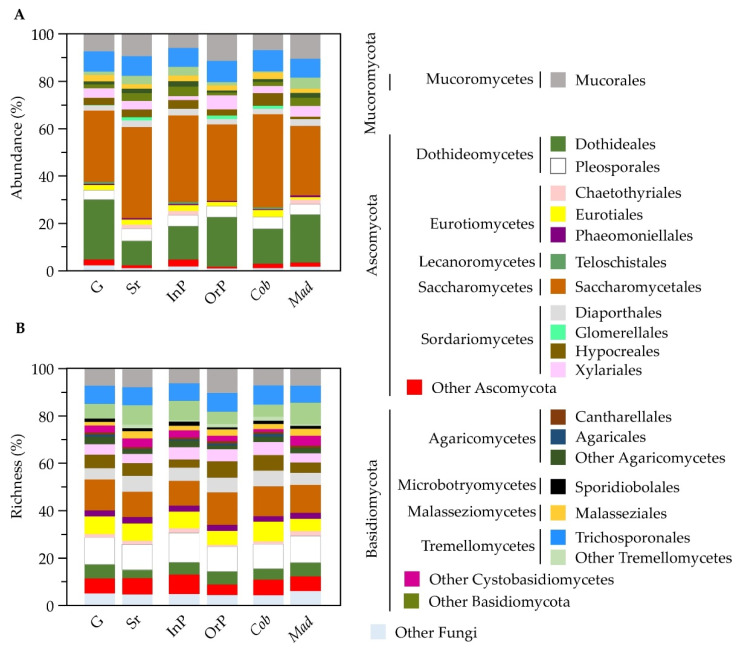
Epiphytic fungal community structure represented by abundance (**A**) and richness (**B**) of identified fungal orders. Fungal communities were found in olives at different maturation stages (green and semi-ripened), obtained from orchards with distinct production systems (integrated and organic production) and collected from different olive cultivars (*Cobrançosa* and *Madural*). Results are presented as the sum of ASVs found for each condition. ‘Other Fungi’ refers to those orders with less than 0.5% of overall abundance/richness.

A total of 1565 fungal ASVs were identified from the olive episphere, most belonging to the Ascomycota phylum (962; 72%), followed by Basidiomycota (439; 17%) and Mucoromycota (95; 9%) phyla (Figure S3). Many ASVs were identified up to family (72%), genera (43%) or species (12%), but more than 27% remained to be identified at family level (Tables S1 and S3). The class Saccharomycetes was the most abundant (35% of total reads), followed by Dothideomycetes (23%), Tremellomycetes (11%), and Sordariomycetes (10%) (Figure S3A). These were also the richest classes, representing 11%, 17%, 15% and 16% of the total fungal ASVs, respectively (Figure S3B). Although the Eurotiomycetes class represented 12% of all identified ASVs, this class was not particularly abundant at olives surface (4% of total reads). This work is the first report where Saccharomycetes was the most abundant detected class in olives, using a metabarcoding approach. This may be due to the fact that specific fungi are more prone to develop (higher relative abundance) in certain cultivars or conditions. For example, Saccharomycetales have been described as being highly abundant in the cv. *Cobrançosa* leaves and branches compared to other cultivars [38]. Indeed, we also found a greater abundance of Saccharomycetales in olives from the cv. *Cobrançosa* (39%) than from the cv. *Madural* (29%), as well as a higher prevalence in semi-ripened olives (38%) in contrast with green olives (30%) (Figure 2A, Table S3). In any case, Saccharomycetales were always the most abundant order in olive epiphytic communities, regardless of olive maturation stage, production system and cultivar. When comparing the fungal composition of olives from different conditions, an almost similar richness in fungal orders profile was found regardless of the microbial community provenance (Figure 2B). However, slight differences were still found regarding olive maturation stage, production system or olive cultivar.

The most abundant order, Saccharomycetales, has been described as containing potent antagonists of various plant pathogens [48]. In this study, the predominant genera of this order was *Candida* (34% of Saccharomycetales reads, corresponding to 12% of total reads), followed by *Kazachstania* (26%, 9%), *Saccharomyces* (12%, 4%), and *Debaryomyces* (8%, 3%) (Figure S3B, Table S3), all known for presenting members with biocontrol activity against plant fungal pathogens [48]. Interestingly when a functional analysis of detected fungal taxa was performed, a higher abundance of beneficial ASVs was detected in specific conditions (olives from cv. *Cobrançosa* and in semi-ripened olives; Figure S4).

### 3.3. Discriminant Fungal Epiphytes in Olives from Different Conditions

**Figure 3:**
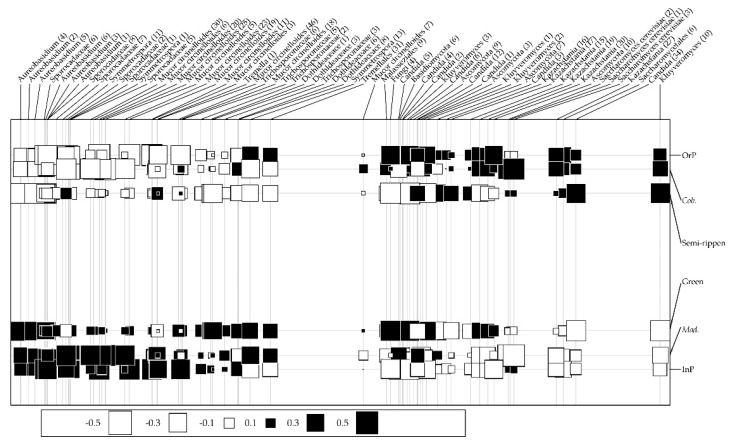
Co-inertia factorial map showing positive (■) and negative (□) relationships between epiphytic fungal ASVs, regarding different maturation stages (green vs. semi-ripened), production systems (integrated vs. organic), and cultivars (*Cobrançosa* vs. *Madural*). Symbol sizes represent the degree of relatedness between variables and fungi. The x-axis represents degree of ASV relationship with all variables: those ASV at x-axis ends are the ones that are most associated with studied variables. The distance between variables (y-axis) represents their contribution towards discrimination of fungal community structures.

Among all identified ASVs, few genera accounted for the majority of reads. The most representative genera were *Aureobasidium* (16% of the total sequences), followed by *Candida* (12%), *Kazachstania* (9%), and *Mucor circinelloides* (8%) (Figure S3B, Table S3). Indeed, the high abundance of *Aureobasidium* was expected because it has been abundantly found in olive tree aerial organs [36]. According to a random forest analysis, these same genera revealed to be important to distinguish the fungal communities from different olive maturation stages, production systems and cultivars (Figure S5). According to their Gini coefficient values, 66 ASVs were selected as the most important to discriminate between fungal communities (all displaying a Gini coefficient higher than 0.032), the most discriminant genera being represented by several ASVs (Table S4). While *Aureobasidium* (4 ASVs) and *Kazachstania* (2 ASVs) were the most important taxa for differentiating the communities from distinct olive maturation stages, Sporocadaceae spp. (2 ASVs) and *Mucor circinelloides* (2 ASVs) were important to distinguish production systems, and *Saccharomyces cerevisiae* (3 ASVs) and *Mucor circinelloides* (2 ASVs) for olive cultivars (Figure S5). In order to further explore the set of fungal genera/species that are associated with olives obtained from each condition (maturation stage, production system and olive cultivar) and evaluate the contribution of these different factors to determine the structure of the epiphytic fungal community, a co-inertia analysis was performed (Figure 3). *Aureobasidium* spp. and Sporocadaceae spp. were positively correlated with green olives, olives from integrated production or from the cv. *Madural*, while Saccharomycetales spp. (*Kluyveromyces*, *Candida*, *Kazachstania* and *Saccharomyces* genera) were positively correlated with semi-ripened olives and those obtained from organic production and the cv. *Cobrançosa*. These Saccharomycetales genera have been reported to comprise effective biocontrol agents against phytopathogenic agents [48]. Although Sporocardaceae spp. have been described as playing multiple functional roles (endophytes, plant pathogens or saprobes, [49]), *Aureobasidium* has been extensively studied as a biocontrol agent (e.g., [50]), even against the causal agents of olive diseases (anthracnose, [51]; Verticillium wilt, [52]). These results suggest that a beneficial fungal community is developed under the studied production systems.

## 4. Conclusions

This work included the most comprehensive assessment of epiphyte fungi associated with olives in different conditions, which has never been performed to date. Although differences in richness were detected, in particular when considering distinct olive management practices and maturation conditions, the fungal composition was similar among production systems and cultivars. Fungal composition among maturation stages revealed to have dissimilarities, in which 5% of epiphyte variation could be attributed to olive maturation. This variation might be attributed to the relevant differences in the most abundant classes (higher Saccharomycetes abundance in semi-ripened olives and lower Dothideomycetes abundance in green olives). Most epiphytes could have arisen from other factors that were not the focus of this work (season, wind speed and temperature [41]), which explains the high number of low abundant reads. However, when focusing on the most discriminant ASVs for each olive condition, the olive maturation stage, production system and cultivar affected the structure of epiphytic assembling. The discriminant power and higher abundance of Saccharomycetales spp. in the cv. *Cobrançosa* (that presents higher tolerance to anthracnose disease in comparison to the cv. *Madural*) suggest a relation of the presence of Saccharomycetales spp. (mainly *Kluyveromyces*, *Candida*, *Kazachstania*, and *Saccharomyces* genera) with lower susceptibility to anthracnose. The set of identified fungal taxa that could control olive disease development should be studied in the near future.

## Figures and Tables

**Table 1:** Number of fungal taxa (*S*) and estimated alpha diversity indices (Simpson’s Index (1-*D*) and Shannon index (*H’*)) in the episphere of olive fruits, considering subsampled dataset. Results are represented by the mean value of different replicates ± SD. Significant differences at *p* ≤ 0.05 between conditions (maturation stages, production systems and cultivars) were determined by ANOVA followed by Tukey’s test (*p* ≤ 0.05). Significant differences among olives from distinct production systems within the same cultivar/maturation stage are denoted by *, whereas those among different olive maturation stages within the same cultivar/production system are underlined. Significant differences were not detected among cultivars.

			Diversity Indexes		
			*S*	1-*D*	*H*’
Green	Organic production	cv. *Madural*	195 ± 79 *	0.970 ± 0.008	4.261 ± 0.387
		cv. *Cobrançosa*	147 ± 61	0.957 ± 0.019	3.909 ± 0.424
	Integrated production	cv. *Madural*	75 ± 39 *	0.917 ± 0.044	3.055 ± 0.630
		cv. *Cobrançosa*	77 ± 28	0.942 ± 0.042	3.480 ± 0.587
Semi-ripen	Organic production	cv. *Madural*	110 ± 61	0.945 ± 0.032	3.587 ± 0.697
		cv. *Cobrançosa*	131 ± 53	0.957 ± 0.021	3.831 ± 0.524
	Integrated production	cv. *Madural*	164 ± 45	0.966 ± 0.023	4.166 ± 0.470
		cv. *Cobrançosa*	97 ± 63	0.941 ± 0.026	3.430 ± 0.595

## Data Availability

Raw data used in this work were deposited in NCBI Sequence Read Archive (http://www.ncbi.nlm.nih.gov/sra (accessed on 24 January 2022)) under BioProject number PRJNA798094.

## Supplementary Materials

The following supporting information can be downloaded at: https://www.mdpi.com/article/10.3390/microorganisms10020376/s1, Figure S1: Rarefaction curves for fungal epiphytic community, Table S1: Number of reads obtained by Illumina MiSeq metabarcoding of *ITS2* DNA samples, Figure S2: Non-metric multidimensional scale (NMDS) plots of fungal epiphyte communities, Table S2: Analysis of similarity (ANOSIM) of microbial communities evaluated in olives, Table S3: Abundance and taxonomic distribution of identified fungal ASVs, Figure S3: Taxonomic distribution (Krona graphics) of ASV abundance (A) and richness (B), Figure S4: Epiphyte functional groups present in olives from each condition, Figure S5: Ranking of relative importance of each ASV, Table S4: Taxonomic distribution of the most discriminant ASVs for distinguishing the epiphyte fungal communities in olives.
