# Supplementary material for: Olive Fungal Epiphytic Communities Are Affected by Their Maturation Stage
Source: Microorganisms. 2022 Feb 5;10(2):376. doi: 10.3390/microorganisms10020376 (PMC8879224; doi:10.3390/microorganisms10020376)
Supplement: Supplementary file 1 [file microorganisms-10-00376-s001.zip › microorganisms-1551801-Supplementary.pdf]

Supplementary material

Olive Fungal Epiphytic Communities are Affected by Their Maturation Stage

Joana Castro [1], Daniela Costa [1], Rui M. Tavares [1], Paula Baptista [2] and Teresa Lino-Neto [1]

Table S1. (Excel file) Number of reads obtained by Illumina MiSeq metabarcoding of *ITS2* DNA samples from olive fruits at different maturation stages (green or semi-ripen olives), obtained from orchards with different management (organic or integrated production) and from distinct olive cultivars (cv. *Cobrançosa* vs. *Madural*). The processing of raw dataset is fully described in Material and Methods section. Classified sequences correspond to those fungal sequences before dataset subsampling. The taxonomic classification of identified fungal ASVs (after subsampling to 3,526 sequences) is given. 1-PM1, 1-PM3 and 1-AM2 samples were not amplifiable. 2-PC3 sample was excluded after subsampling. Due to the reduced number of classified fungal sequences, 2-PC3 sample was removed for further analysis.

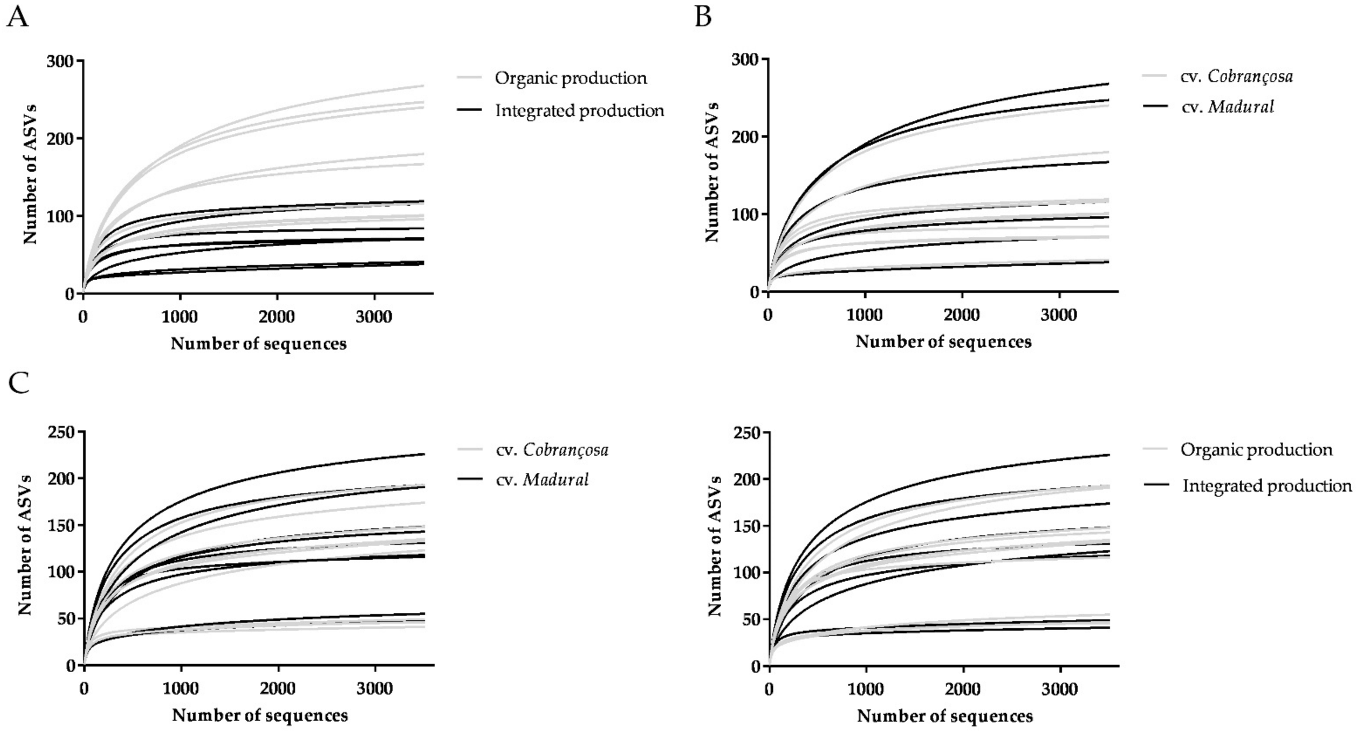

Figure S1. Rarefaction curves for fungal epiphytic community present in green olive fruits (A, B) and semi-ripen olive fruits (C, D). Samples from different production systems are differently highlighted in A and C, while samples from different cultivars are displayed in different colors in B and D.

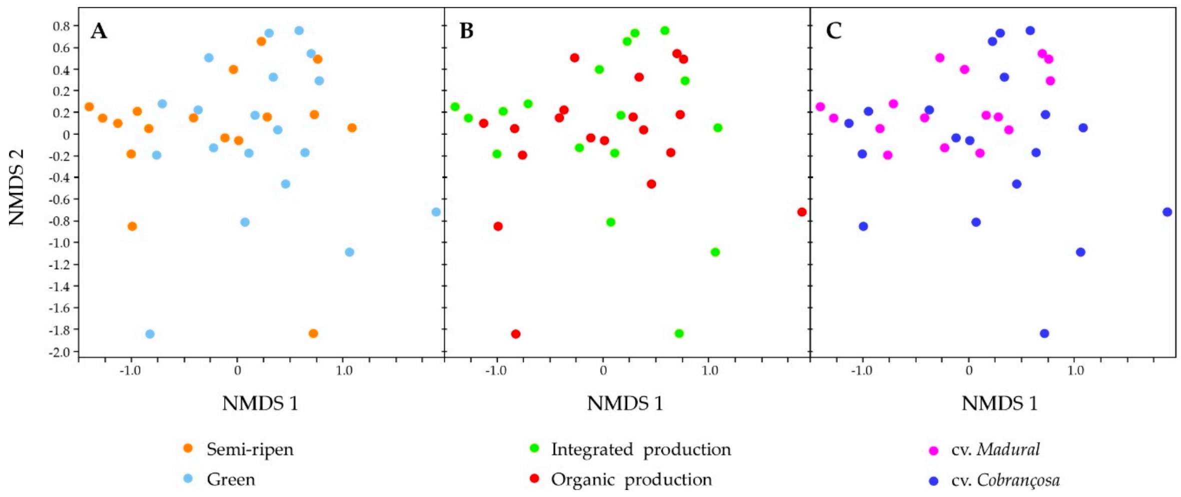

Figure S2. Non-metric multidimensional scale (NMDS) plots of fungal epiphyte communities (2D stress value = 0.190) using the Bray-Curtis dissimilarity coefficient. Microbial communities are highlighted according to the olives they came from: maturation stage (A), production system (B) and cultivar (C).

Table S2. Analysis of similarity (ANOSIM) of microbial communities evaluated in olives from different maturation stages, production systems or cultivars using the Bray-Curtis coefficient. Presented values correspond to *R* value. Statistical significances were not detected.

Microbial communities origin			Maturation stage	Production system	Olive cultivar
Green	Organic production	cv. *Madural*	0.18	0.18	0.06
		cv. *Cobrançosa*	0.06	-0.01	-
	Integrated production	cv. *Madural*	0.16	-	0.05
		cv. *Cobrançosa*	-0.003	-	-
Semi-ripen	Organic production	cv. *Madural*	-	-0.07	-0.13
		cv. *Cobrançosa*	-	-0.11	-
	Integrated production	cv. *Madural*	-	-	0.20
		cv. *Cobrançosa*	-	-	-

Table S3. (Excel file) Abundance and taxonomic distribution of identified fungal ASVs, detected by Illumina MiSeq metabarcoding of *ITS2*. Taxonomic distribution is displayed for all identified taxa levels.

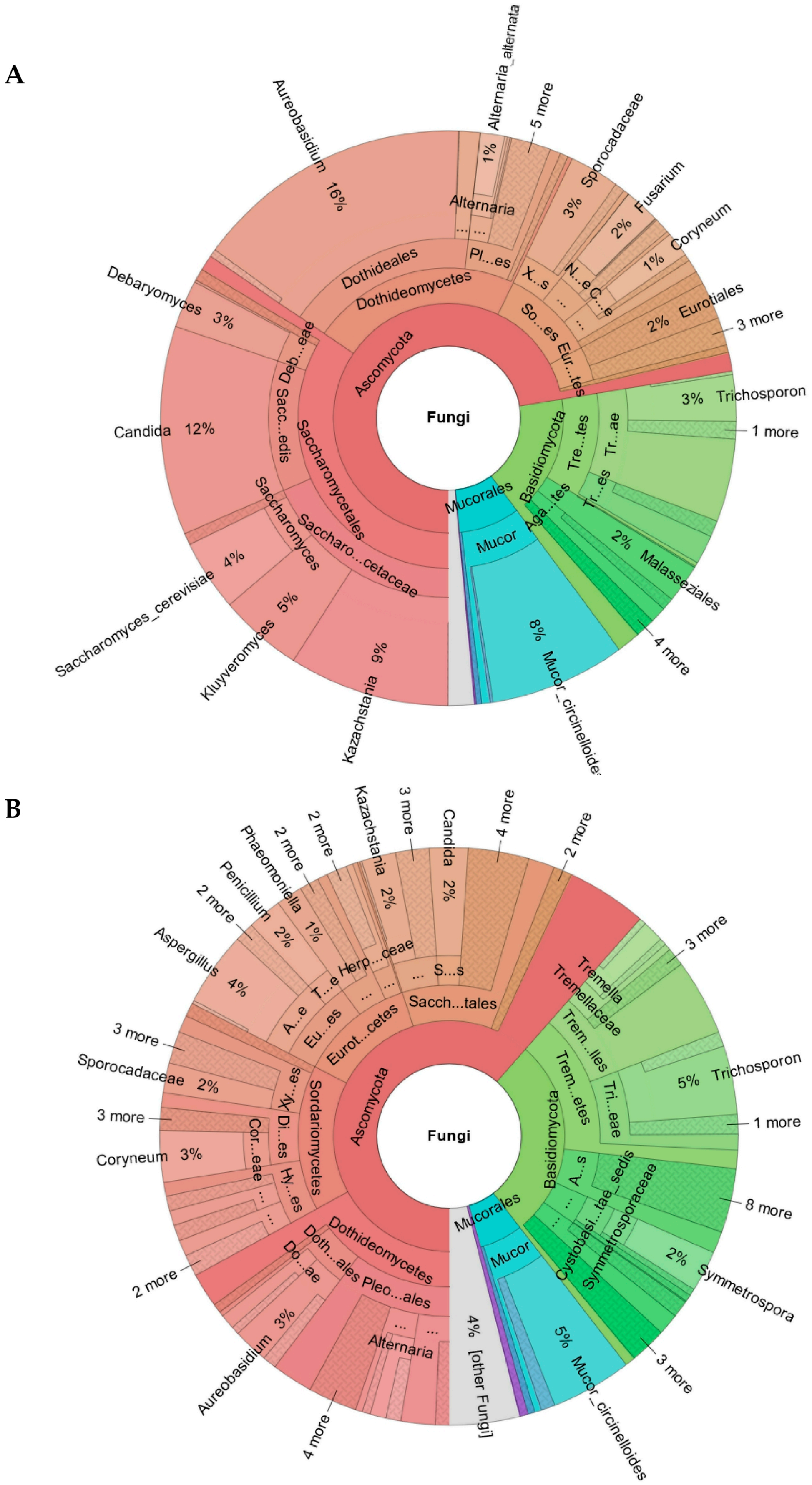

Figure S3. Taxonomic distribution (Krona graphics) of ASV abundance (A) and richness (B), detected in the olive episphere. Data correspond to all fungal classified sequences.

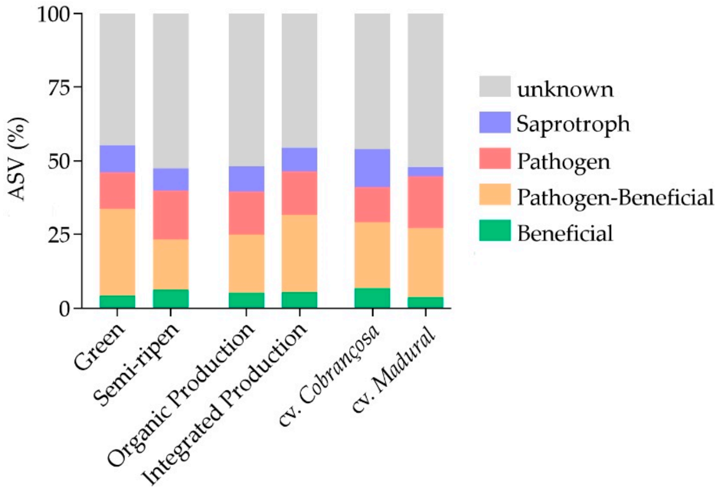

Figure S4. Epiphyte functional groups present in olives from each condition. The ecological role of each ASVs was determined using FUNGuild tool. Functional groups displaying ASVs with possible pathogenic role are identified as 'pathogen' and those with possible beneficial role are identified as 'beneficial'.

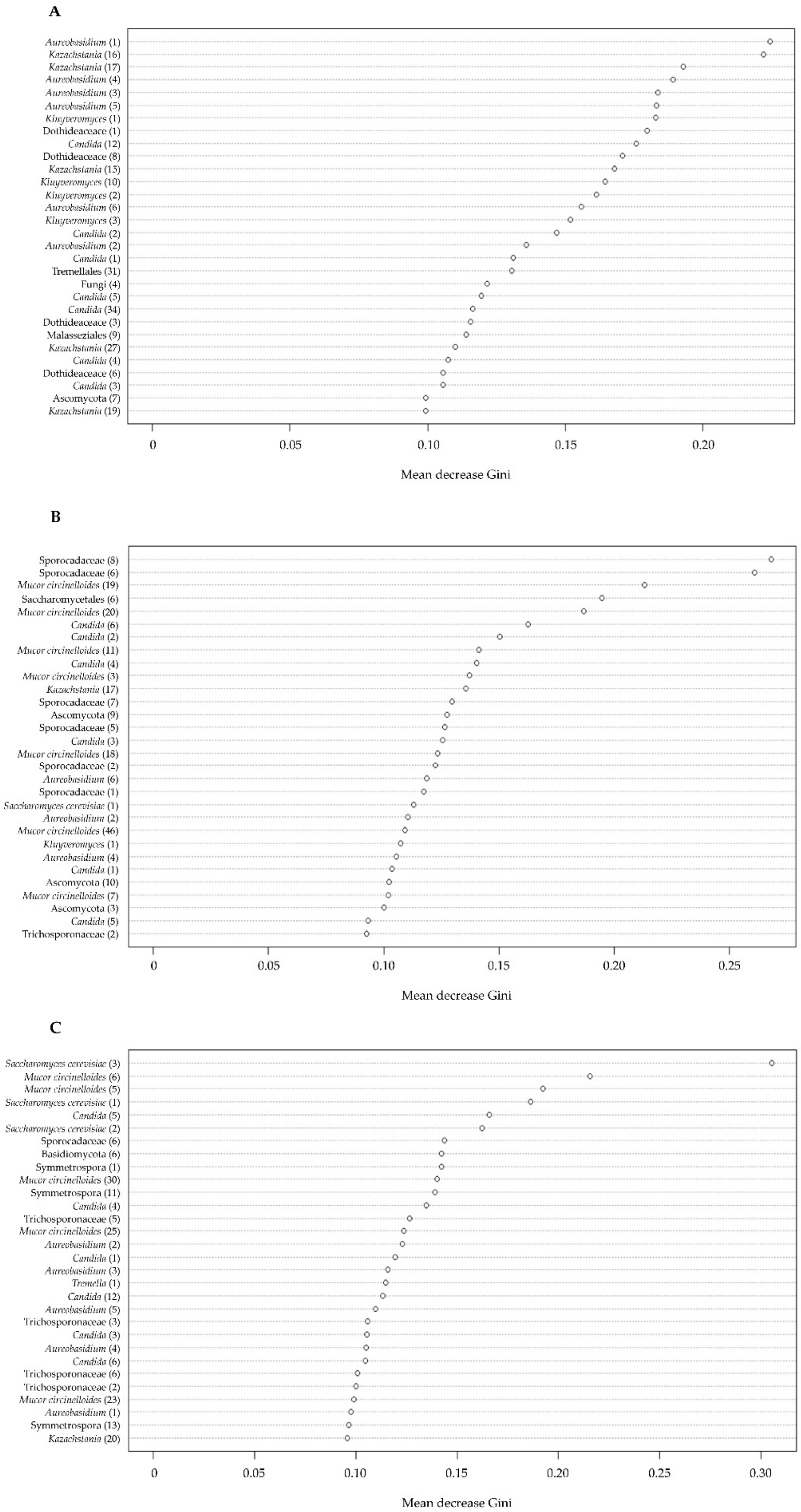

Figure S5. Ranking of relative importance of each ASV for distinguishing maturation stages (semi-ripen and green olives), production systems (integrated production and organic production) and cultivars (cvs. *Madural* and *Cobrançosa*), considering epiphytic fungal communities. Mean Decrease Gini value measure the importance of ASV, with highest values representing the best predictors.

Table S4. Taxonomic distribution of the most discriminant ASVs for distinguishing the epiphyte fungal communities in olives, considering the olive maturation stages, production systems and cultivar. Most taxa were represented by several ASVs, which is indicated by the 'No. of ASVs'. The maximal Gini coefficient of each taxa is displayed.

**Taxonomic classification**				**No. ASVs**	**Max Gini**		
					**Maturation stage**	**Production system**	**Olive cultivar**
Ascomytota	Dothideomycetes						
		Dothideales					
			Aureobasidium	6	1.968e-01	1.524e-01	2.012e-01
			Dothideaceae	4	1.323e-01	2.906e-02	3.181e-02
	Saccharomycetes						
		Saccharomycetales					
			Candida	8	1.990e-01	1.592e-01	1.373e-01
			Kazachstania	6	3.395e-01	1.030e-01	1.050e-01
			Kluyveromyces	4	2.195e-01	7.047e-02	1.178e-01
			Saccharomyces cerevisiae	3	5.675e-02	1.022e-01	2.302e-01
			Saccharomycetales sp.	1	6.422e-03	9.855e-02	2.120e-02
	Malasseziomycetes						
		Malasseziales					
			Malasseziales sp.	1	5.120e-02	2.760e-02	7.901e-02
	Sordariomycetes						
		Xylariales					
			Sporocadaceae spp.	6	6.863e-02	2.419e-01	1.012e-01
	Cystobasidiomycetes						
		Cystobasidiomycetes Incertae sedis					
			Symmetrospora	3	2.047e-02	4.682e-02	1.771e-01
	Other Ascomycota			4	1.120e-01	9.020e-02	1.434e-02
Basidiomycota	Tremellomycetes						
		Tremellales					
			Tremella	1	5.116e-03	2.436e-02	5.292e-02
			Tremellales sp.	1	7.461e-02	1.175e-01	1.256e-02
			Trichosporonaceae spp.	4	1.049e-01	1.052e-01	1.794e-01
	Other Basidiomycota			1	1.441e-02	5.650e-02	1.074e-01
Zygomycota	Zygomycetes						
		Mucorales					
			Mucor circinelloides	12	7.517e-02	1.702e-01	2.193e-01
